# From Structure-Function Analyses to Protein Engineering for Practical Applications of DNA Ligase 

**DOI:** 10.1155/2015/267570

**Published:** 2015-10-05

**Authors:** Maiko Tanabe, Yoshizumi Ishino, Hirokazu Nishida

**Affiliations:** ^1^Central Research Laboratory, Hitachi Ltd., 1-280 Higashi-koigakubo, Kokubunji, Tokyo 185-8601, Japan; ^2^Department of Bioscience and Biotechnology, Graduate School of Bioresource and Bioenvironmental Sciences, Faculty of Agriculture, Kyushu University, 6-10-1 Hakozaki, Higashi-ku, Fukuoka-shi, Fukuoka 812-8581, Japan

## Abstract

DNA ligases are indispensable in all living cells and ubiquitous in all organs. DNA ligases are broadly utilized in molecular biology research fields, such as genetic engineering and DNA sequencing technologies. Here we review the utilization of DNA ligases in a variety of *in vitro* gene manipulations, developed over the past several decades. During this period, fewer protein engineering attempts for DNA ligases have been made, as compared to those for DNA polymerases. We summarize the recent progress in the elucidation of the DNA ligation mechanisms obtained from the tertiary structures solved thus far, in each step of the ligation reaction scheme. We also present some examples of engineered DNA ligases, developed from the viewpoint of their three-dimensional structures.

## 1. Introduction

DNA ligases are critical DNA replication and repair enzymes; they have been widely used in molecular biology and biotechnology applications, such as cloning and next-generation DNA sequencing [1, 2]. DNA ligases catalyze the joining of adjacent 3′-hydroxyl and 5′-phosphorylated DNA termini in duplex DNA. All DNA ligases accept nicked dsDNA and homologous, cohesive ends as substrates, although the minimum length of the overlap required for efficient ligation varies widely. Some ligases, most notably T4 DNA ligase, also accept fully base-paired (blunt end) substrates for* in vitro* ligation reactions.

DNA ligases share a common mechanism and a high degree of structural similarity with other members of the nucleotidyltransferase superfamily, including RNA ligases and RNA-capping enzymes [3]. Like other nucleotidyltransferases, DNA ligases utilize an ATP molecule to activate the enzyme, and thus DNA ligases are prepared to react with DNA substrates [4].

The details of each step in the DNA ligation reaction sequence have been clarified by many efforts performed by numerous researchers. Above all, Shuman's group has made extensive contributions toward the elucidation of the DNA ligation mechanisms [5]. We describe the exemplified DNA ligation schemes in particular, in the second section of this review.

Since the discovery of DNA ligase, this enzyme has been widely utilized in several types of molecular biology and biotechnology applications. DNA ligase is frequently utilized in combination with restriction enzymes in recombinant DNA experiments. The enzyme has been applied in DNA sequencing methods in diverse ways, by taking advantage of the fact that DNA ligase does not require dNTPs as substrates for its function, since large amounts of dNTPs in solution, a general situation in DNA polymerase reactions, sometimes inhibit effective measurements and cause misinterpretations. DNA ligase is also utilized in analytical methods for protein-protein interactions, as developed by Landegren et al. [6]. These methods are widely applied for quick and effective* in situ* investigations of protein-protein interactions [7]. We will describe the details of the utilization of DNA ligase in several aspects of genetic engineering technology and molecular and cell biology in the third section.

Since the first report of the X-ray crystal structure of the ATP-dependent DNA ligase from bacteriophage T7 [8], the crystallographic analyses of the archaeal and eukaryotic ATP-dependent DNA ligases were subsequently reported during the first decade of the new century [9–11]. The ATP-dependent DNA ligases from Archaea and Eukarya comprise three domains, but, surprisingly, the relative arrangements of the three domains were completely different from each other in these reports [9–11], reflecting the dynamic domain motion in the 3-step ligation sequence. Based on these structural transitions in the ligation sequence, several attempts toward improving the enzymatic reaction have been made by mutating the enzymes. In the fourth and the fifth sections, we summarize the structural information about DNA ligase and the mutation-based improvements of its enzymatic efficacy.

## 2. Basis of the DNA Ligase Reaction

The Gellert, Lehman, Richardson, and Hurwitz laboratories discovered DNA ligases in 1967 and 1968 [12–15]. By joining the 3′-OH and 5′-phosphate termini (nicks) to form a phosphodiester bond, DNA ligases play an important role in maintaining genome integrity. They are essential for DNA replication and repair in all organisms [16, 17]. Furthermore, DNA ligases have long been a critical reagent in the development of molecular cloning and many subsequent aspects of DNA biotechnology.

**Figure 1:**
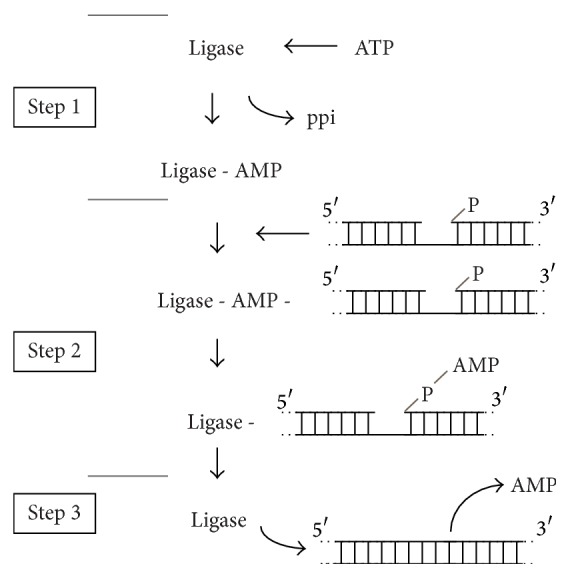
Schematic diagram of the three-step reaction catalyzed by ATP-dependent DNA ligases. The three-step reaction catalyzed by DNA ligase (Ligase) results in the serial transfer of AMP (adenosine 5′-monophosphate) to an active site lysine (step 1) and then to the 5′-PO_4_ end of DNA (step 2). During step 3, the 3′-OH end of a second DNA strand attacks the 5′-PO_4_, to release AMP and generate the ligated DNA product.

All DNA ligation reactions entail sequential nucleotidyltransfer steps [18]. The reaction mechanism can be divided into three distinct catalytic events: the first (step 1), the activation of the enzyme through the covalent addition of AMP to the conserved catalytic lysine of the ligase, accompanied by the release of PPi or nicotinamide mononucleotide from the cofactor (ATP or NAD^+^); the second (step 2), the binding of the ligase-adenylylate to the substrate DNA and the transfer of AMP from the ligase to the 5′-phosphoryl group of the nick on the DNA; and the third (step 3), the formation of the phosphodiester-bond with the concomitant release of free AMP from the DNA-adenylylate intermediate (Figure 1). All three chemical steps depend on a divalent cation. The DNA ligases are grouped into two families, according to the cofactor dependence for the reaction. ATP-dependent DNA ligases are found in viruses, bacteria, Eukarya, and Archaea [18, 19], whereas NAD^+^-dependent DNA ligases are primarily present in bacteria and entomopox viruses [19, 20].

**Figure 2:**
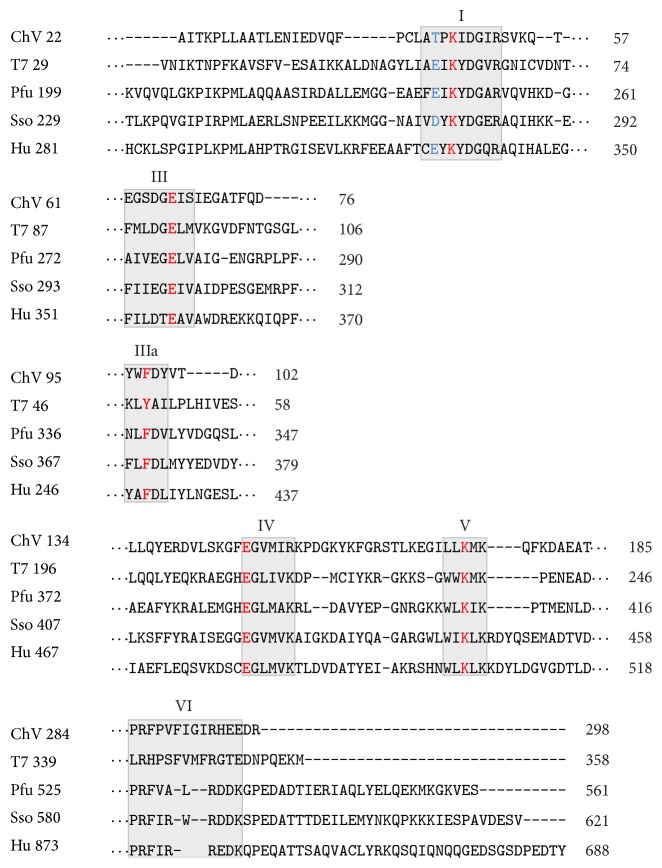
Conserved sequence elements define a superfamily of covalent nucleotidyltransferases. Six collinear sequence elements, designated as motifs I, III, IIIa, IV, V, and VI, are conserved in capping enzymes and ligases. The amino acid sequences are aligned for the DNA ligases from* Chlorella* virus (ChV), bacteriophage T7 (T7),* Pyrococcus furiosus* (Pfu),* Sulfolobus solfataricus* (Sso), and human (Hu). The number of intervening amino acid residues is indicated. Conserved residues are highlighted in red (nucleotidyltransferases) and blue (DNA and RNA ligases).

Sequence analyses of DNA ligases revealed that they share six motifs (I, III, IIIa, IV, V, and VI), which are also conserved in the nucleotidyltransferase superfamily members, including RNA ligase [21], tRNA ligase [22], and mRNA-capping enzymes [23], except for motif VI. In the ATP-dependent DNA ligases, these six motifs align well among the enzymes from viruses, bacteriophages, Archaea, and Eukarya, without long gaps or insertions (Figure 2). The sequences of the ATP-dependent DNA ligases showed that the amino acids in motifs I, III, IIIa, IV, V, and VI contact the substrates, AMP or DNA. The important roles of the individual amino acids in these motifs were verified by alanine scanning mutational studies and confirmed as essential for one or more steps of the ligation pathway [24]. For example, motif I (K**x**DG**x**R) contains the lysine that becomes covalently linked to AMP in the first step of the ligase reaction [25, 26]. Furthermore, the arginine and lysine side chains in motif VI (RxDK) orient the PPi leaving group apically relative to the attacking motif I lysine, during step 1 enzyme nucleotidylation reaction [27, 28].

## 3. Utilization of DNA Ligases in Genetic Research Technologies

The* in vitro* manipulation of DNA has been broadly applied to studies in molecular genetics, microbiology, immunology, and oncology and also to practical uses in clinical assays [29]. This technology was developed with DNA-related enzymes, which were discovered by research on the molecular mechanisms of the replication and repair of DNA. DNA replication is initiated by DNA primase, which synthesizes small RNA primers that are subsequently elongated by DNA polymerases [30]. Based on its primer extension activity, a DNA polymerase can be utilized in the polymerase chain reaction (PCR), which amplifies a single copy of DNA by several orders of magnitude in a brief* in vitro* reaction [31]. In addition, DNA ligase plays a role in joining Okazaki fragments during lagging strand maturation [32]. Based on its activity to join two DNA strands* in vitro*, DNA ligase has been contributing to recombinant DNA technology, for example, DNA cloning [33]. In addition, DNA ligase is broadly utilized for ligase-mediated mutation detection methods and DNA sequencing.

### 3.1. Oligonucleotide Ligation Assay (OLA)

DNA ligase can only join two DNA strands when they are perfectly hybridized to a complementary DNA sequence. Even a single base pair mismatch between two strands significantly decreases the efficiency of the strand joining reaction [34]. In 1988, the Oligonucleotide Ligation Assay (OLA) was developed, as a useful method to detect the genotype of the target DNA by utilizing this characteristic [6, 35]. This was the first example of the development of a method for gene analysis by using DNA ligase.

**Figure 3:**
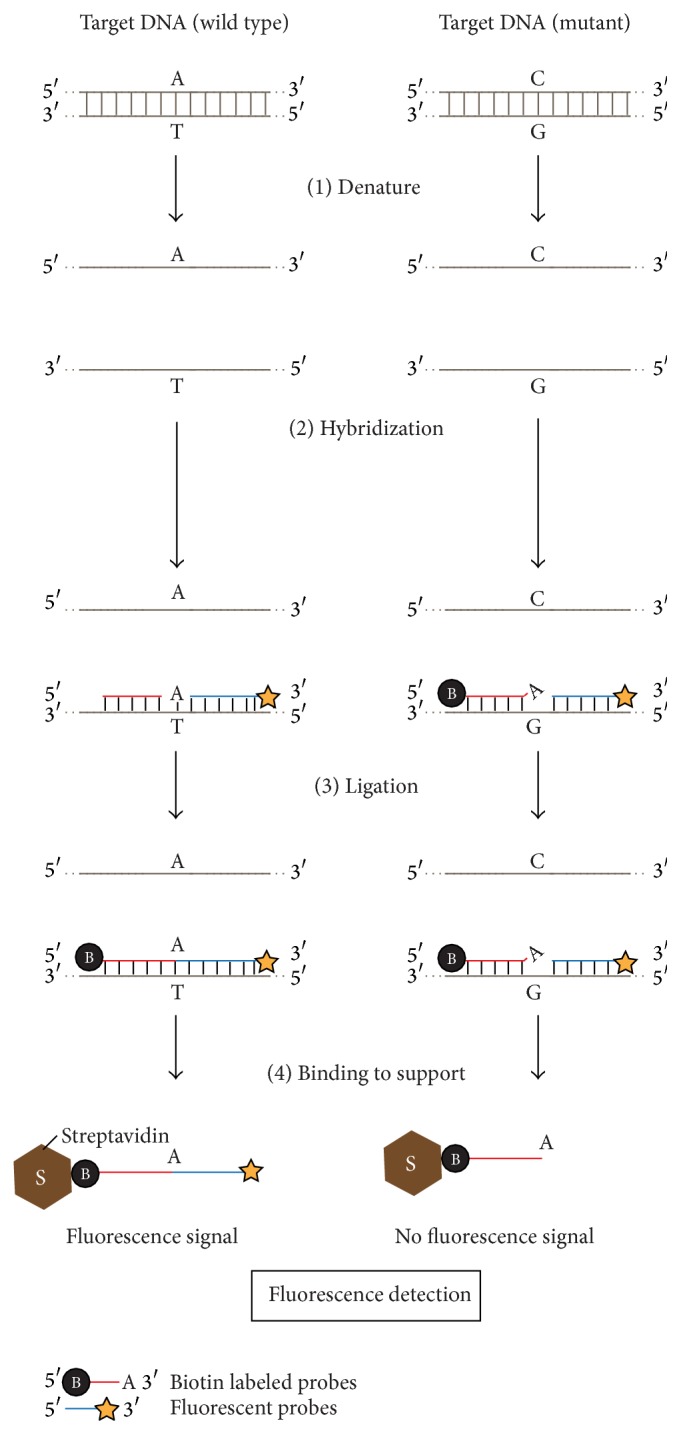
Schematic diagrams for the Oligonucleotide Ligation Assay (OLA). DNA fragments from the normal type and the mutant type (at the single nucleotide polymorphism (SNP) site) are shown as reaction templates. (1) Denature: samples are heated to 85–95°C for one to several minutes to denature (separate into single strands) the target DNA. (2) Hybridization: two oligonucleotide probes, 5′-biotin labeled probes and 3′-fluorescent (or radiolabeled) probes, which are complementary to the normal-type target, hybridize to the target DNA fragment. (3) Ligation: adjacent probes that are perfectly complementary to the target (left) are connected by DNA ligase. (4) Binding to support: the fluorescently labeled ligation samples are immobilized to streptavidin and detected by fluorography only if ligated to biotinylated oligonucleotides that can be bound to streptavidin on a solid support (left).

OLA consists of two steps. First, the two probes to be hybridized with the target DNA at the sequence of interest must lie directly adjacent to one another. In this case, one probe must contain a reporter group, which is either ^32^P- or fluorescently labeled, and the other must include a recognition group for immobilization, such as a biotin group, which could be captured by streptavidin immobilized on a solid support. Second, the neighboring probes that are perfectly hybridized to the target DNA at the sequence of interest are joined by DNA ligase. Then, the ligation signal is subsequently captured by streptavidin and detected by autoradiography or fluorography (Figure 3). This assay takes advantage of the enzymatic accuracy in two events, the hybridization of the probes and the joining by DNA ligase, resulting in the reliable analysis of the clinical genotype.

A breakthrough in OLA for practical use occurred in 1990, when PCR was coupled to the assay prior to the ligation reaction (PCR-OLA) [36]. PCR-OLA amplification only for the target DNA enhanced the sensitivity of the assay, enabling the nonradioactive detection of the OLA results. Recently, a sensitive, specific, and high-throughput OLA was developed for the detection of genotypic human immunodeficiency virus type 1 (HIV-1) resistance to a Food and Drug Administration-approved protease [37]. This report revealed that OLA can be used for the detection of the HIV-1 genotype (wild type or mutant) as a genetic diagnosis method.

### 3.2. DNA Ligase-Mediated Cycling of the Ligation Reaction

**Figure 4:**
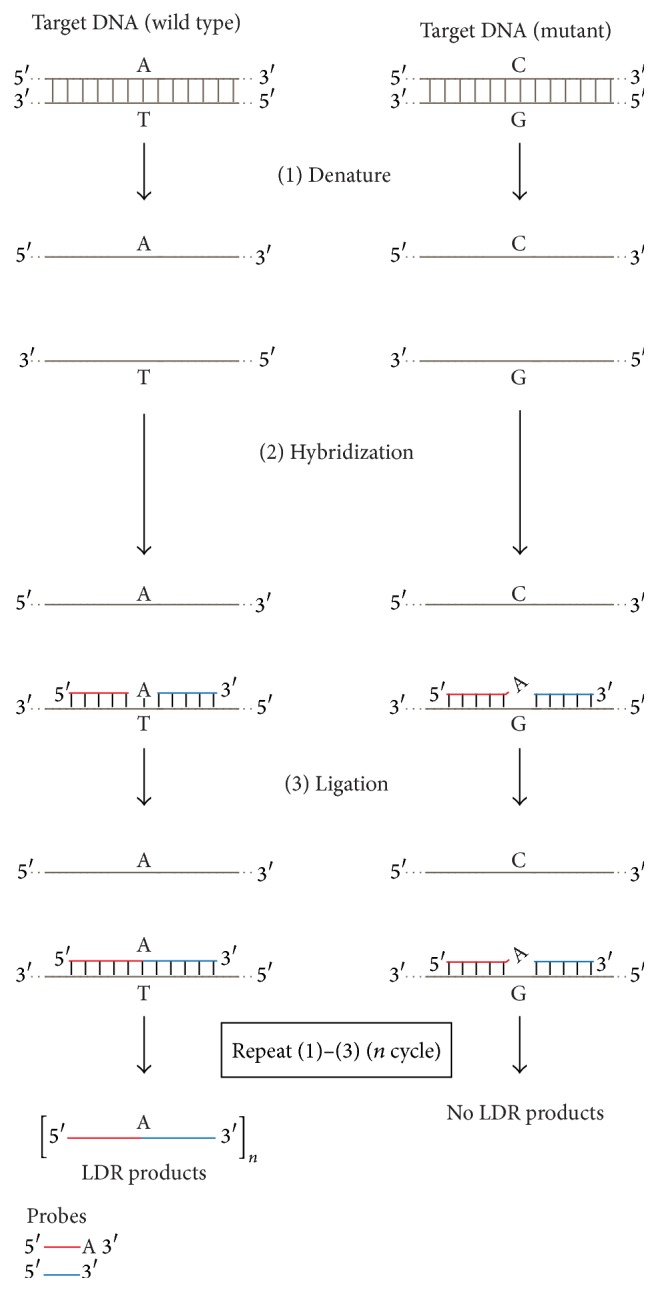
Schematic diagrams for the Ligation Detection Reaction (LDR) processes. DNA fragments from the normal type and the mutant type (at the single nucleotide polymorphism (SNP) site) are shown as reaction templates. (1) The denaturation process is the same as in Figure 3 (1). (2) Hybridization: two oligonucleotide probes, complementary to the normal-type target, hybridize to the target DNA fragment. (3) Ligation: adjacent probes that are perfectly complementary to the target (left) are connected by DNA ligase, and thermocycling linearly amplifies the amount of product formed. In the case of the mutant type template, the presence of a single-base mismatch at the junction inhibits the ligation, and therefore no ligated LDR probes are formed (right).

**Figure 5:**
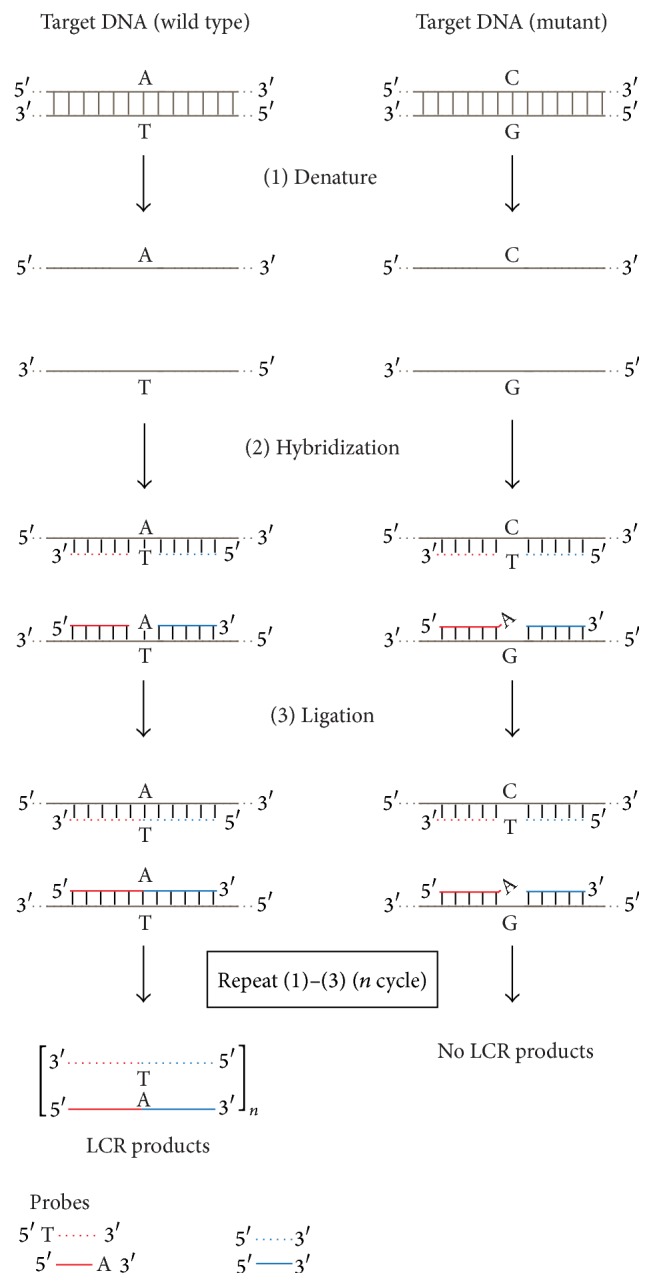
Schematic diagrams for the Ligation Chain Reaction (LCR) and Ligation Amplification Reaction (LAR) processes. (Here, we describe LCR.) DNA fragments from the normal type and the mutant type (at the single nucleotide polymorphism (SNP) site) are shown as reaction templates. (1) The denaturation process is the same as in Figure 3 (1). (2) Hybridization: four oligonucleotide probes, complementary to the normal-type target, hybridize to the target DNA fragment, and (3) Ligation: adjacent probes that are perfectly complementary to the target (left) are connected by DNA ligase. Ligated LCR probes from the first round of the ligation become the targets for the subsequent round using another probe set (dotted lines) complementary to the first set. Thus, the amount of ligated LCR probes increases exponentially. In the case of the mutant type template, the presence of a single-base mismatch at the junction inhibits the ligation, and therefore no ligated LCR probes are formed (right).

Further spreading of DNA ligase-mediated technologies was achieved by cycling the ligation reaction, like PCR, using a thermostable DNA ligase, which became available commercially in 1990. The thermostable DNA ligase enables the cycling OLA reaction. OLA reaction method with thermal cycling procedure developed by using thermostable DNA ligase is often specifically termed Ligation Detection Reaction (LDR) (Figure 4). Through repeated denaturation, annealing, and ligation, the target signal is amplified linearly [38–40]. Ligation Chain Reaction (LCR) [38, 39, 41] and Ligation Amplification Reaction (LAR) [42] are also performed by repeated cycles of heat denaturation of a DNA template containing the target sequence, annealing of probes, and ligation processes. After annealing the first set of two adjacent oligonucleotide probes to the target DNA sequence in a unique manner, a second set of complementary oligonucleotide probes that hybridize to the sequence opposite to the target DNA sequence is applied (Figure 5). Thereafter, a thermostable DNA ligase will covalently link each pair of adjacent probes that are completely hybridized at the junction of the adjacent probes. Through the repeated sequential processes, including denaturation, annealing, and ligation, the target signal is amplified exponentially. These methods require a thermostable DNA ligase to allow the ligation to occur under temperature conditions that prevent mismatches from hybridizing to form acceptable substrates. The thermostable DNA ligase made this assay useful for DNA-based diagnostic tests for inherited diseases in clinical laboratories. The ligation-mediated detection of point mutations by thermostable DNA ligase is now used to detect hepatitis B virus (HBV) mutants [43–45], colon tumor microsatellite sequence alterations [46], the mutation responsible for bovine leukocyte adhesion deficiency [47], AZT-resistance HIV mutants [48], mutations in the* ras* family of oncogenes [49], extended spectrum *β*-lactamase resistance in bacteria [50], and ganciclovir-resistant cytomegalovirus mutants [51].

### 3.3. Padlock Probe and Rolling-Circle Amplification (RCA)

**Figure 6:**
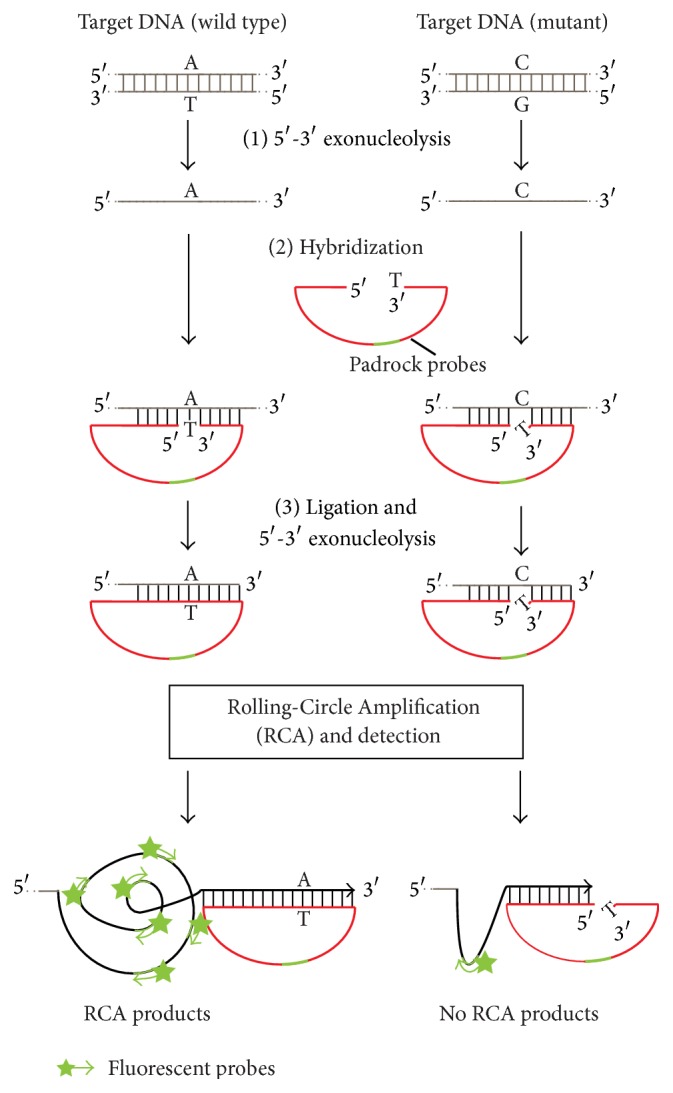
Schematic representation of the target-primed Rolling-Circle Amplification (RCA) of circularized padlock probes. (1) 5′–3′ exonucleolysis: the target DNA is restriction digested 3′ of the target sequence and irreversibly made single stranded by strand-specific 5′–3′ exonucleolysis. (2) Hybridization: padlock probes (red) with the tag sequence (green) are hybridized to their target sequences. (3) Ligation & 5′–3′ exonucleolysis: adjacent padlock probes that are perfectly complementary to the target (left) are connected by DNA ligase, thus locking the probe onto the target molecule. After ligation, the RCA is initiated by the Phi29 DNA polymerase, by turning the target molecule into a primer through the 3′–5′ exonucleolysis of any 3′ end protruding beyond the padlock probe hybridization site. The padlock probe then serves as the template for DNA synthesis. The RCA product (black) is detected through the hybridization of fluorescently labeled probes (green) to tag sequences specific for the padlock probe. Arrowheads indicate 3′ ends of the RCA reaction.

Padlock probes and RCA are alternative methods for detecting known sequence variants in DNA and particularly for detecting single nucleotide polymorphisms, by using a thermostable DNA ligase. The padlock probe has target recognition sequences situated at both the 5′- and 3′-ends, connected by an intervening sequence that can include the sequence for detection. When the probe is hybridized to a target sequence, the two ends are brought adjacent to each other, and then a thermostable DNA ligase covalently joins the two ends and circularizes the probe. This circularized probe is wound around the target strand in a manner similar to a padlock, driven by the helical nature of the double-stranded DNA [52] (Figure 6). This probe is used for the localization of signals in* in situ* analyses. For example, a padlock probe was able to detect repeated alphoid sequences in metaphase chromosomes in a living cell, demonstrating its utility for* in situ* studies [53–55]. RCA is also known as a simple and efficient isothermal enzymatic process that utilizes strand-displacement DNA and RNA polymerases, like Phi29, Bst, and Vent exo-DNA polymerases for DNA and T7 RNA polymerase for RNA, to generate long single stranded DNAs and RNAs containing tens to hundreds of tandem repeats that are complementary to the circular template [56–58]. The padlock principle has been combined with RCA for mutation detection, in which a primer annealing to the linker region initiates rolling circle replication (Figure 6), because the padlock approach alone is not sufficient to detect single nucleotide differences in a single copy gene* in situ* [59–61]. Point mutations in the cystic fibrosis transmembrane conductance regulator (CFTR), p53, BRCA1, and Gorlin syndrome genes were visualized in interphase nuclei and DNA fibers by RCA [62]. These results demonstrated that ligase-mediated mutation detection methods coupled with RCA are able to reveal single nucleotide differences in a single cell. The ability to detect mutations in a cellular milieu has important implications for cancer research and diagnosis.

**Figure 7:**
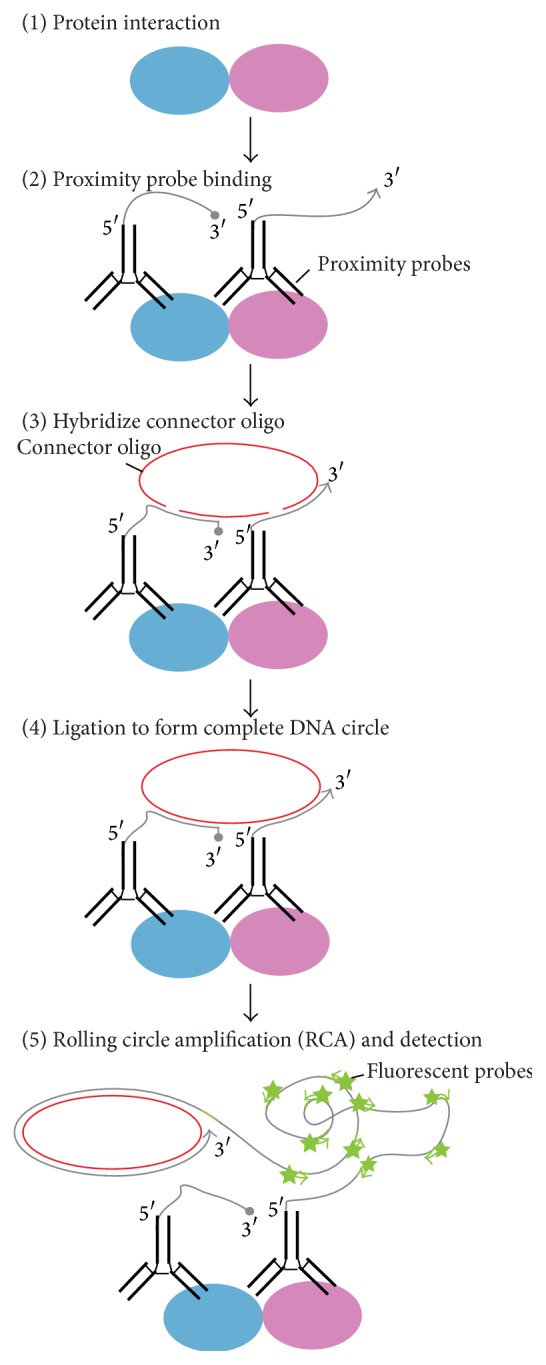
Schematic representation of* in situ* Proximity Ligation Assay (PLA). (1) Protein interaction: the complex is formed between target proteins (light blue and pink). (2) Proximity probe binding: antibodies (black) with individual proximity probes (glay) bind to each of the target protein complexes. (3) Hybridization of connector oligo-DNAs: target protein complex serves to template the hybridization of connector oligo-DNAs (red). (4) Ligation to form complete DNA circle: adjacent connector oligos that are perfectly complementary to the target template are connected by DNA ligase. (5) Rolling Circle Amplification (RCA) and detection: after ligation, the RCA is initiated by the Phi29 DNA polymerase, by turning the target molecule primed by one of the proximity probes. The RCA product is detected through the hybridization of fluorescently labeled probes (green). Arrowheads indicate 3′ ends, and roundheads means 3′ ends modified with 2′* O*-methyl RNA.

In another aspect of the RCA with ligation methods, Proximity Ligation Assay (PLA) is known for one of the potent techniques for detecting individual proteins or protein complexes* in situ*, by using antibodies with attached DNA strands that participate in ligation and subsequent RCA reactions [63]. PLA can be used for detecting reactions in which identification of target molecules depends on two recognition events. Formation of a proper target complex results in the formation of a circular DNA strand by exogenously added DNA ligase. This circular DNA is used to template a locally restricted RCA reaction, which generates an elongating ssDNA rolling-up in a ball that can be detected when hybridized with fluorescence-labeled probes [7]. The binding to a target interacting complex by two different antibodies with attached oligonucleotides individually (referred to as proximity probes) is followed by the addition of two more oligonucleotides that are then ligated into a circular DNA strand by the function of DNA ligase. The circular DNA strand is templated by the oligonucleotides attached to antibodies (Figure 7). Next, one of the antibody-bound oligonucleotides is utilized as a primer of the succeeding RCA reaction, resulting in the generation of an ssDNA rolling circle product. The rolling circle is covalently attached to one of the proximity probes. A 60 min RCA by Phi29 DNA polymerase results in a 1000-fold amplification of the 100 nt DNA circle, producing an around 100 kb rolling circle product [63]. The rolling circle product is then visualized by hybridization of fluorescence-labeled complementary oligonucleotide detection probes* in situ*.

### 3.4. Next-Generation DNA Sequencing by Using DNA Ligase

**Figure 8:**
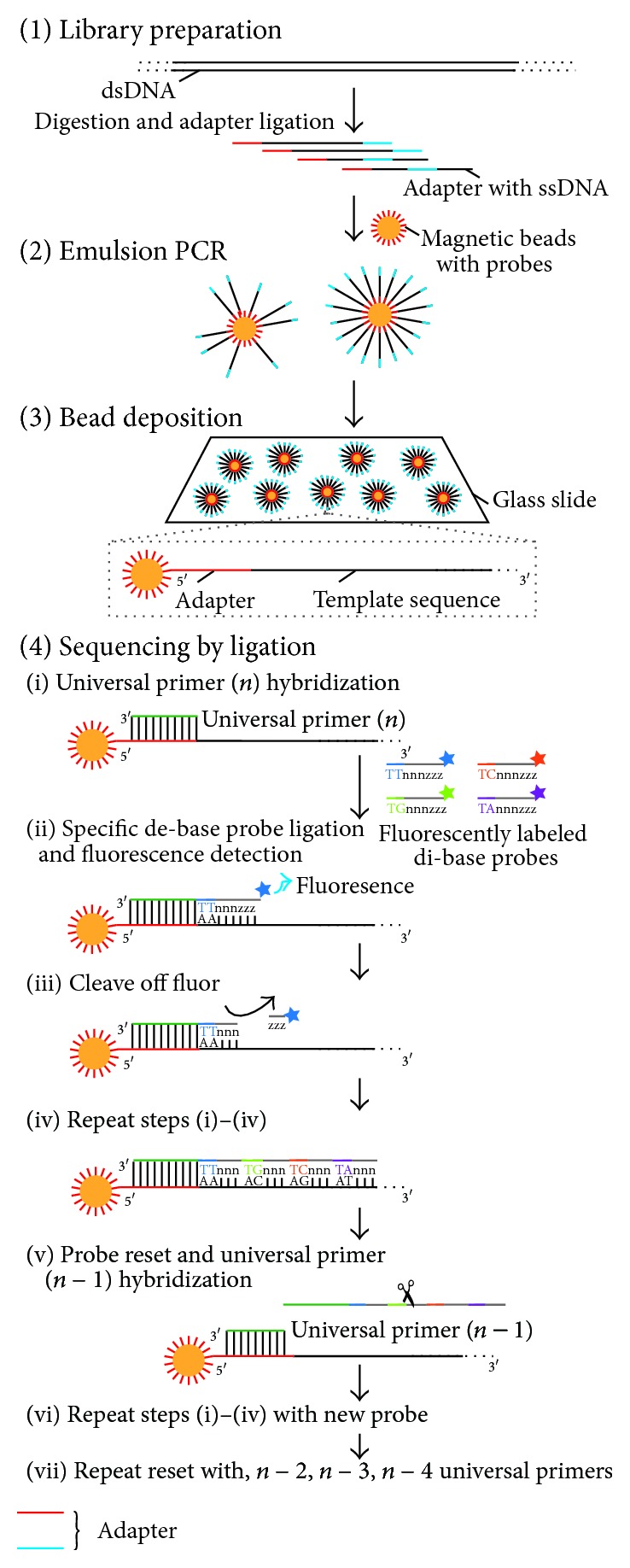
The ligase-mediated sequencing approach of the Sequence by Oligonucleotide Ligation and Detection (SOLiD) sequencer (Life Technologies). (1) Library preparation: two different adapters are ligated to sheared genomic DNA. (2) Emulsion PCR: emulsion PCR is conducted using magnetic beads to generate “bead clones,” in which each contains a single nucleic acid species. (3) Bead deposition: the beads are then attached to the surface of a glass slide. (4) Sequencing by ligation: ligase-mediated sequencing begins by annealing a universal primer to the shared adapter sequences on each amplified fragment (i), and then DNA ligase is provided along with specific fluorescently labeled 8-mers, in which the two bases at the 3′ end of the probe are encoded by the attached fluorescent group. Each ligation step is followed by fluorescence detection (ii), after which a regeneration step removes the bases from the ligated 8-mer (including the fluorescent group) (iii), and concomitantly prepares the extended probe for another round of ligation (iv–vii). Since each fluorescent group on a ligated 8-mer identifies a two-base combination, the resulting sequence reads can be screened for base-calling errors versus either true polymorphisms or single base deletions, by aligning the individual reads to a known high-quality reference sequence.

Recently, Next Generation Sequencing (NGS) technologies, such as the 454 FLX pyrosequencer (Roche) [64], Illumina genome analyzer (Illumina) [65], and Sequence by Oligonucleotide Ligation and Detection (SOLiD) sequencer (Life Technologies) [66], have revolutionized genomic and genetic research. Although these platforms are quite diverse, in terms of their sequencing biochemistry and sequence detection, their workflows are conceptually similar to each other. The array is prepared by random fragmentation of DNA, followed by* in vitro* ligation of common adaptor sequences by DNA ligase. DNA fragments with adapters are amplified by DNA polymerase and are detected by each platform. The detection platforms rely on sequencing by DNA synthetic methods, that is, the serial extension of primed templates. The enzyme driving the DNA synthesis can be either a DNA polymerase or a DNA ligase. The 454 FLX Pyrosequencer and Illumina analyzer use DNA polymerase methods with pyrosequencing [67] and reversible dye terminator technology, respectively [68]. In contrast, SOLiD uses DNA ligase and a unique approach to sequence the amplified fragments [69]. A universal primer complementary to the adaptor sequence is hybridized to the array of amplicons. Each cycle of sequencing involves the ligation of a degenerate, fluorescently labeled 8-mer probe set (Figure 8). The octamer mixture is structured, and the type of nucleotide (A, T, G, or C) at the specific position within the 8-mer probe set correlates with the type of fluorescent label. After the ligation of the 8-mer probes, images are acquired in four channels, resulting in the effective collection of sequencing data for the 3′ end positions of the probes across all template-bearing beads. Then, the octamer is chemically cleaved between positions 3 and 4, to remove the fluorescent label. Progressive rounds of octamer ligation enable the sequencing of every 5th base (e.g., bases 1, 6, 11, and 16 of the template strand). After several cycles of probe ligation reactions, the extended primer is denatured to reset the system. Subsequent iterations of this process can be applied to a different set of positions (e.g., bases 0, 5, 10, and 15 of the template strand), by using different mixtures of octamers in which the nucleotides at the different position are correlated with the label colors. An additional feature of this platform involves the use of two-base encoding, an error-correction scheme in which two adjacent bases, rather than a single base, are correlated with the label. Each base position is then queried twice (in a set of 2 bp interrogated in a given cycle) and thus miscalls can be more readily identified [70].

## 4. Structural Transition of the DNA Ligase in the Reaction Sequence

In Section 2, we showed that the sequence alignments clearly revealed several homologous regions among ATP-dependent DNA ligases and RNA capping enzymes, indicating the presence of five conserved motifs (I, III, IIIa, IV, and V) in these proteins. This fact suggests that the nucleotidyltransferase superfamily members, including ATP-dependent DNA ligases, may share a similar protein fold and a common reaction mechanism. Next, we will present a series of crystal structures of ATP-dependent DNA ligases and describe the structural and functional insights into the mechanisms of the enzymes.

### 4.1. Structural Studies of ATP-Dependent DNA Ligase

**Figure 9:**
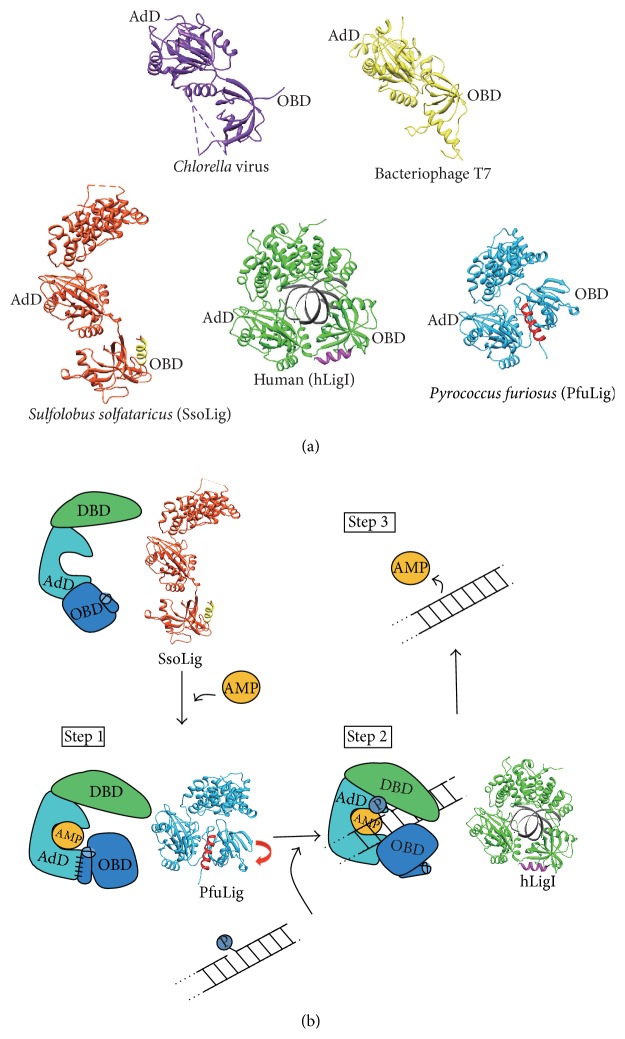
Crystal structures of ATP-dependent DNA ligases. (a) The viral and bacterial ATP-dependent DNA ligases from* Chlorella* virus (left, blue) and bacteriophage T7 (right, yellow) are two-domain ligases. These ligases revealed the common structures of two distinct domains, designated as the adenylylation domain (AdD) and the oligonucleotide/oligosaccharide-binding-fold domain (OBD), which are jointly called the catalytic core domains. In these two structures, the catalytic core domains adopted an open form. The three-domain structure of DNA ligase is characteristic of the archaeal (*Sulfolobus solfataricus*: SsoLig (orange, left) and* Pyrococcus furiosus*: PfuLig (blue, right)) and eukaryotic (human: hLigI (green, center)) DNA ligases. The AdD contains most of the catalytic residues and is assisted by two flanking domains that also bind to DNA, the N-terminal DNA-binding domain (DBD) and the C-terminal OBD. Each C-terminal helix is highlighted in yellow (SsoLig), magenta (hLigI), and red (PfuLig). The figure was prepared using Chimera [105]. (b) Schematic diagram of the structure-based ligation mechanism. Before and after the ligation reaction, DNA ligase adopts the extended conformation, as expected from the SsoLig crystal structure. In step 1, the closed conformation of the catalytic core domains at the carboxyl terminus in PfuLig creates a small compartment, which holds a covalently bound AMP molecule. In step 2, the interactions with the substrate DNA must be stabilized for the enzyme to adopt a compact conformation, as seen in the crystal structure of hLig1 bound to the nicked-DNA. Finally, the ligation of the DNA strands and the subsequent release of AMP and DNA strands from DNA ligase occur during step 3.

ATP-dependent DNA ligases show considerable variation in their molecular sizes, which range from 41 kDa (bacteriophage T7) [71] to 102 kDa (human DNA ligase I (hLigI)) [72]. Despite this wide size variation, it is clear from the sequence alignments that the smaller enzymes constitute a common core structure that is conserved across all ATP-dependent ligases [73]. The crystal structures of the ATP-dependent DNA ligases, such as bacteriophage T7 DNA ligase (T7Lig) complexed with ATP [8, 74] and* Chlorella* virus DNA ligase (ChvLig) with covalently bound AMP [75], have been solved (Figure 9(a), top). These DNA ligases adopt a common architecture of two distinct domains: the adenylylation domain (AdD) and the oligonucleotide/oligosaccharide-binding-fold domain (OBD), which are jointly called the catalytic core domains. The catalytic core domains are the minimal entity for the nick-joining activity, as seen in the ChvLig and T7Lig enzymes [8, 75]. The AdD contains the catalytic lysine residue that forms a covalent bond with AMP, which is derived from the substrate ATP. The Lys238 and Lys240 residues of T7Lig (Lys188 and Lys186 of ChvLig) within the AdD are essential for the adenylylation and nick sealing activities [76, 77]. The Lys240 residue forms a photo-crosslinking adduct with the 5′-terminal nucleotide of the nick, implying its direct involvement in binding the phosphate of the nick [78]. The OBD is connected to the AdD via the conserved motif V [5, 79] and is similar to other DNA and RNA binding proteins [80, 81]. The OBD is observed in the related nucleotidyltransferase, the mRNA capping enzyme from* Chlorella* virus, which undergoes opened-closed conformational changes during catalysis. The OBD domain of the enzyme was found to move towards AdD and close the nucleotide-binding pocket [80, 81].

In contrast, three crystal structures of large ATP-dependent DNA ligases, which mainly ligate the nicks between Okazaki fragment in DNA replication in Eukarya and Archaea, with an additional N-terminal DNA-binding domain (DBD), have been reported (Figure 9(a), bottom) [9–11, 82, 83]. This extra domain is not essential for hLigI activity* in vitro* [84, 85] but is considered to be crucial for detecting a singly nicked dsDNA [9]. The structures of the two archaeal DNA ligases from* Pyrococcus furiosus* (PfuLig) and* Sulfolobus solfataricus* (SsoLig) were determined in the closed [11] and extended forms [10], respectively. The structure of hLigI in complex with DNA [9] revealed that the enzyme entirely encircles the nicked-DNA. All of these DNA ligases are commonly composed of three domains (DBD, AdD, and OBD) in the sequences from the N to C termini. Although the protein folding of each domain is strikingly similar among the three DNA ligases, the relative domain orientations within each enzyme are quite different.

### 4.2. Structural Transition of DNA Ligase Molecules in Each Reaction Step

**Figure 10:**
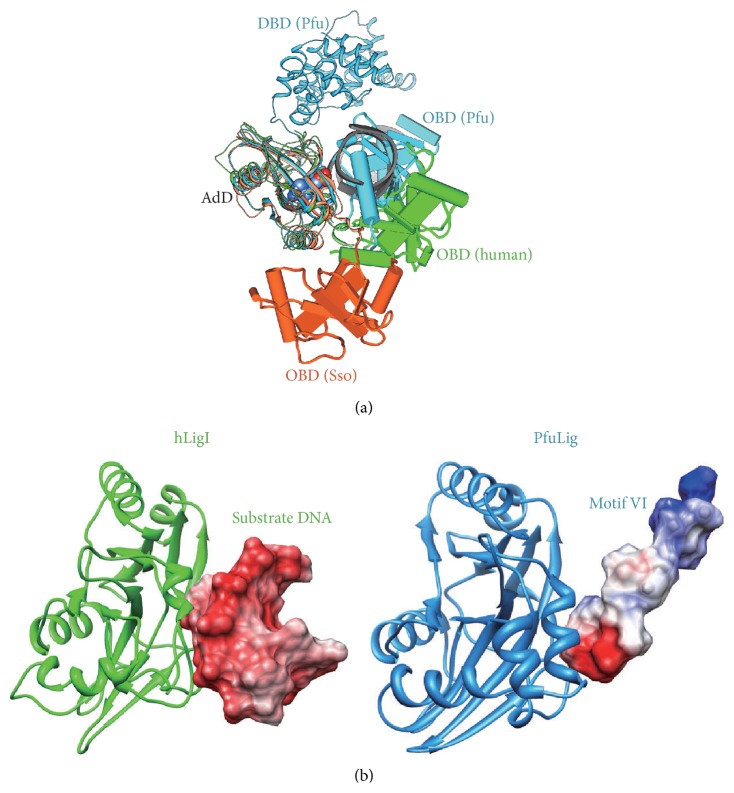
Superimposed ribbon diagrams. (a) Ribbon diagrams of PfuLig (2CFM, blue), hLigI (1X9N, green), and SsoLig (2HIV, orange), in which the AdD from each ligase were maximally overlapped with each other. The DBD from hLigI and SsoLig were omitted, for clarity. The arrangements of the OBD relative to the AdD in each ligase are apparently different from each other. Notably, the OBD from PfuLig is closely bound to AdD and is replaced by the bound-DNA substrate (grey) in hLigI. (b) Superimposed diagrams of adenylation domains from PfuLig and hLigI, flanked by surface representations of motif VI (PfuLig) and the upstream region of the substrate DNA (hLigI), in which the electrostatic distributions (positive charge, blue; negative charge, red) are mapped onto the molecular surfaces. Since the electrostatic distributions on the surfaces nearby the AdD of motif VI and the DNA are both negatively charged, the replacement of motif VI with DNA may easily occur.

Based on the crystal structures described above, a ligation mechanism was proposed, as schematically represented in Figure 9(b). In solution, a DNA ligase partly adopts the extended form, as expected from the SsoLig crystal structure and the small angle X-ray scattering (SAXS) analysis [10]. In the absence of DNA and AMP, the OBD of SsoLig is turned away from the AdD in an open conformation, resembling that seen in the crystal structures of the compact and two-domain (AdD-OBD) DNA ligases from* Chlorella* virus and T7 (Figure 9(a)). The closed conformation of the catalytic core domains, which represents the active conformation for step 1, is observed in the crystal structures of PfuLig, as proposed previously [10, 11]. In step 2, tight binding between the enzyme and the substrate DNA occurs, as revealed by the hLigI–DNA complex crystal structure [9]. These structures depict three different phosphoryl transfer reactions, and the flexible multidomain structure of DNA ligases facilitates the adoption of different enzyme conformations during the course of the reaction (Figure 9(b)) [10]. A superimposition of the AdDs in PfuLig, SsoLig, and hLigI revealed that the arrangements of the OBD relative to the AdD in each ligase are apparently different from each other. Notably, the OBD from PfuLig is closely bound to the AdD and is replaced by the bound-DNA substrate in hLigI (Figure 10(a)). Ribbon diagrams of the AdDs from hLigI and PfuLig, together with the adjacent surface representations of the substrate DNA (hLigI) and motif VI (PfuLig), are shown in Figure 10(b). The electrostatic distribution on the motif VI surface facing AdD is negative, suggesting that motif VI is a molecular mimic of the incoming DNA substrate [11]. This finding indicates that the ligation could be completed simply by the smooth replacement between the substrate DNA and motif VI. Indeed, in the closed structure of PfuLig, motif VI approaches the active site in the absence of DNA, whereas it occupies a position in the region upstream from the nicked DNA in the structure of hLigI.

### 4.3. Interface of the Mandatory Domains (AdD and OBD) for the Enzymatic Reaction

**Figure 11:**
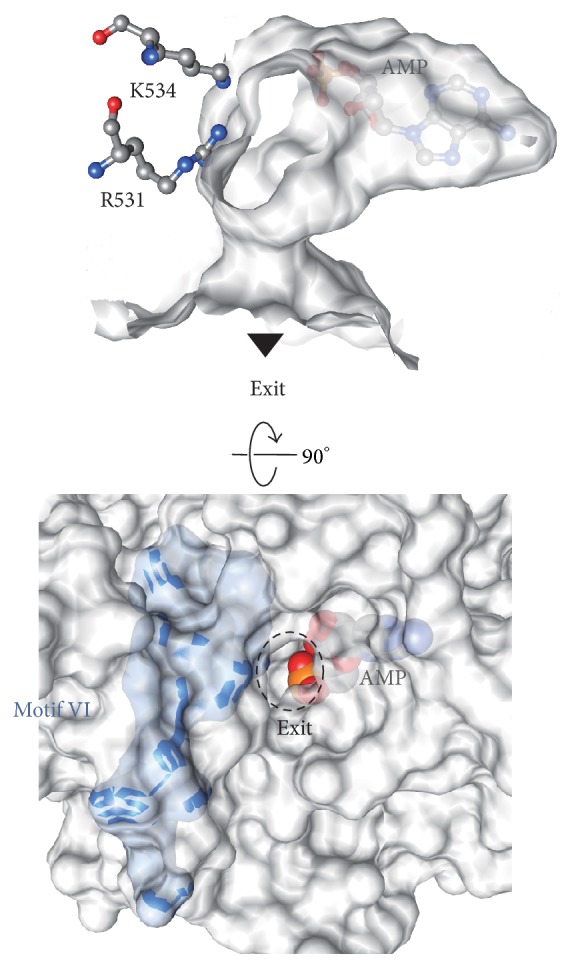
Top: the AMP-binding pocket viewed from the inside of the molecule. A solvent-accessible surface was created without the bound AMP, and then the final refined AMP model was superimposed. The noncovalently bound AMP is trapped within a closed compartment, which conforms well to the shape of the AMP molecule. Bottom: surface representation around the hole in the active site pocket. The surface of motif VI is colored cyan. The bound AMP is depicted as a sphere model. The phosphate (phosphorus, orange; oxygen, red) group is visible through a small “exit.”

In the ATP-dependent DNA ligases, the enzyme captures ATP to adenylylate a Lys residue in the first step, in which motif VI is also indispensable [27]. Motif VI in PfuLig provides several basic residues located around the AMP binding pocket. This electron density contributes to forming the compartment that traps the AMP molecule (Figure 11). In particular, R531 and K534 in motif VI, specific to the DNA ligases in Archaea and Eukarya, contribute to the basic surface within the pocket (Figure 11) [11].

**Figure 12:**
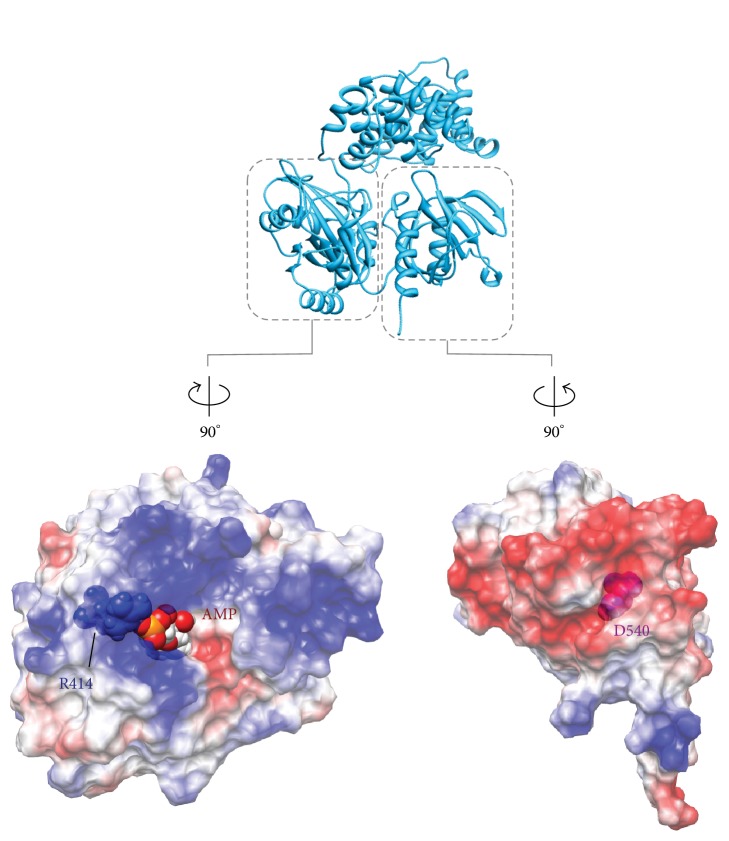
The electrostatic distributions on the interacting surfaces of AdD (bottom, left) and OBD (bottom, right), in which one of the ionic interaction pairs (Arg414 and Asp540) is highlighted as sphere models.

The electrostatic potential distributions of the AdD-OBD interacting surfaces (Figure 12) revealed that the predominant charge distribution on each surface was oppositely charged, and this may be involved in stabilizing the closed conformation of the two catalytic core domains [11]. There is a small exit of the pocket through the closed conformation of the two domains (Figure 11), and the diameter of the exit is about 5.5 Å and could accommodate the PPi molecule but not the AMP moiety. The exit may serve for the spontaneous release of the PPi product. This notion is consistent with the fact that the DNA ligase from* Pyrococcus horikoshii*, which is more than 90% identical to PfuLig, cannot use NAD^+^ as a nucleotide cofactor [86], because the pocket is too small to accommodate the nicotinamide ring to be released from the active site. Presumably, this compact “reaction room” of PfuLig would have been specifically designed to complete the conversion process from ATP to AMP within the closed pocket [11].

### 4.4. Role of the C-Terminal Helix Commonly Observed in the Archaeal and Eukaryotic DNA Ligases

**Figure 13:**
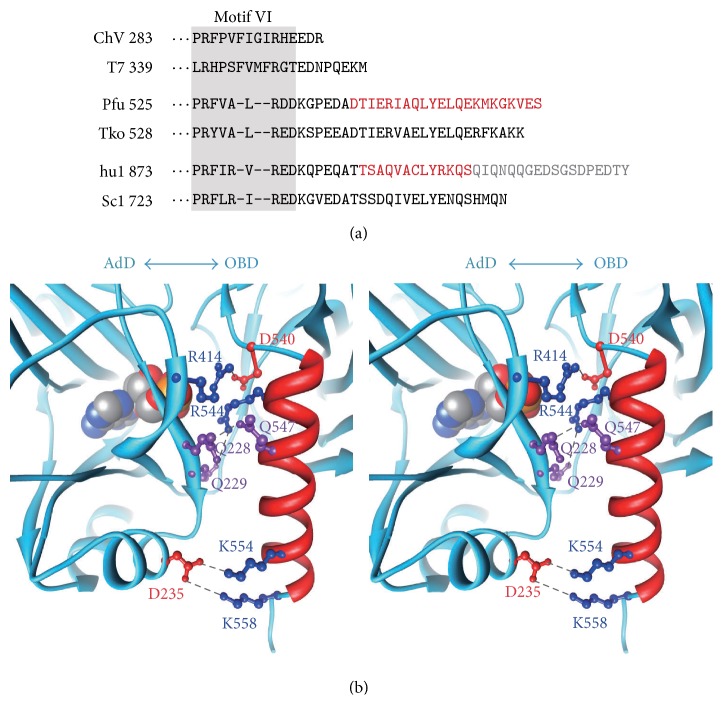
Roles of the C-terminal extension helix. (a) Alignment of the sequences of motif VI and the C-terminal extension. The sequences of the DNA ligases from a virus (ChV,* Chlorella* virus), bacteriophage (T7, bacteriophage T7), Archaea (Pfu,* Pyrococcus furiosus*; Tko,* Thermococcus kodakaraensis*), human (hu1, human DNA ligase I), and yeast (Sc1,* Saccharomyces cerevisiae* DNA ligase I) are aligned. The region for motif VI is shaded, and the extension helix region, determined by the crystal structures of PfuLig and hLigI, is colored red (residues after Q902 of hLigI were not included in the previously reported crystal structure and are colored gray). (b) Stereo diagram of the interface of the AdD and the OBD in PfuLig. Ball-and-stick models represent the amino acid residues involved in the interaction between the two domains: basic, acidic, and polar residues are colored blue, red, and purple, respectively. The C-terminal helix is colored red. The dotted lines indicate the polar or ionic interactions between AdD and OBD.

The overall architectures of the three domains in hLigI and PfuLig are similar to each other (Figure 9(a)), although the sequence identities between the corresponding fragments of hLigI and PfuLig are moderate (32%). The crystal structures and sequence analyses revealed that the eukaryotic and archaeal DNA ligases possess a C-terminal helix shortly after motif VI (Figures 9(a) and 13(a)). The sequence extension harboring the C-terminal helix is conserved among the eukaryotic and archaeal DNA ligases, whereas the ligases from viruses (*Chlorella* virus (ChV)) and bacteriophages (bacteriophage T7 (T7)) lack this long extension (Figure 13(a)). The C-terminal helix is located at the boundary of the AdD and the OBD in the closed structure of PfuLig [11]. In the case of hLigI, this helix is distant from the domain interface, because of the bound DNA substrate (Figure 9(a)). In fact, a structural comparison between the DNA-hLigI complex and PfuLig suggested that the C-terminal helix might play a crucial role in switching from the tightly closed form to the DNA-substrate-bound form. The C-terminal helix of PfuLig connects the AdD and the OBD through five polar or ionic interactions (Figure 13(b)). This feature suggests that the helix might play a critical role in stabilizing the closed conformation of the catalytic core domains during step 1.

## 5. Engineered DNA Ligases with Improved Abilities

As described in Section 3, DNA ligases are critical for many applications in molecular biology. Improvements in the enzymatic ability, such as the nick sealing efficiency or fidelity, will provide better performance in the current methods for ligase-mediated mutation detection and NGS, as described above. Numerous improvements of DNA polymerases have been reported [87–89], whereas fewer are known for DNA ligases. Here we describe some reports on improvements of the enzymatic performances of DNA ligases.

### 5.1. Improvement of the Nick-Sealing Activity by Fusion with the Archaeal DNA Binding Domain

The ATP-dependent DNA ligase from bacteriophage T4 (T4Lig) has evolved to be a nick-sealing enzyme. It can also join double-stranded DNA (dsDNA) fragments with cohesive ends [90]. Furthermore, it is the only commercially available DNA ligase that can join blunt-ended DNA duplexes* in vitro*, in the absence of macromolecular enhancers such as polyethylene glycol [91, 92]. The ligation reaction of cohesive or blunt-ended dsDNA fragments is prerequisite for NGS, which requires the* in vitro* ligation of common adaptor sequences. However, the turnover numbers for the fragment joining reactions of T4Lig are lower than those for nick sealing, and T4Lig is approximately five orders of magnitude less efficient in joining blunt-ended duplexes than nick-sealing [92, 93]. T4Lig is also inefficient for ligating fragments with single base overhangs [94]. The poor fragment joining activity of T4Lig often leads to NGS failures. In order to improve the efficiency of fragment joining by T4Lig, Wilson et al. constructed a variety of chimeras of T4Lig fused with the DNA-binding domains from other enzymes [92]. This mutational strategy was inspired by a previous report, in which the genetic fusion of a sequence of a nonspecific archaeal DNA binding protein (Sso7d from* Sulfolobus solfataricus*) to Pfu DNA polymerases resulted in considerably increased processivity and improved performance in polymerase chain reaction (PCR) amplifications [88]. The fusion of T4Lig with Sso7d showed a 60% increase in performance over T4Lig in the cloning of a blunt-ended fragment [92].

### 5.2. Improvement of the Fidelity of Thermostable DNA Ligase by Site-Directed Mutagenesis

The specificity of DNA ligase is exploited in LCR and LDR analyses to distinguish single base mutations associated with genetic diseases. The ligation fidelity of T4Lig is improved by the presence of spermidine and by high salt and low ligase concentrations [6, 34]. However, the increased fidelity of T4Lig by adjusting the reaction conditions is not sufficient for the ideal detection of SNPs [6, 34, 95, 96]. The thermostable DNA ligases from* Thermus aquaticus* (Taq) and* Thermus thermophilus* (Tth) exhibited far greater fidelity than that reported for T4Lig [39, 97]. The ligation reactions at the higher temperature prevented mismatched hybridizations in the substrates. Luo et al. reported the further improvement of the fidelity of TthLig by site-directed mutagenesis. The fidelity of DNA polymerases was decreased by site-directed mutagenesis at the motif associated with primer-template binding or the exoIII motif [98–100]. However, the exoIII motif mutants, also known as “antimutator” strains, showed increased fidelity in which the balanced activities of the polymerizing and 3′ → 5′ exonuclease reactions might improve the overall fidelity [99–102]. Two mutant ligases, K294R and K294P, were identified with fidelities increased by ~4-fold and 11-fold, respectively, besides retaining their nick sealing activities [97].

### 5.3. Structure-Based Mutational Study of an Archaeal DNA Ligase towards Improvement of the Ligation Activity

**Figure 14:**
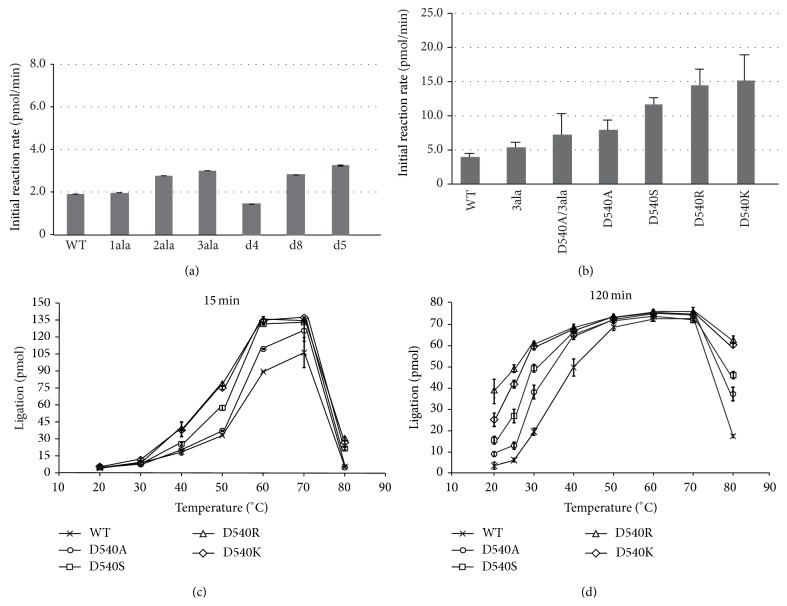
Nick-joining activities of the wild type and mutant proteins. (a) Initial reaction rates for the wild type (WT), K558A (1ala), K554A/K558A (2ala), Q547A/K554A/K558A (3ala), C-terminal 4 residue-deleted mutant d4 (open circles), d8 (open squares), and d15 (open triangles) enzymes, represented by time course data. (b) Initial reaction rates for WT, 3ala, D540A/3ala, D540A, D540S, D540R, and D540K. (c) Nick-joining activities of Asp540 mutants at each temperature for 15 min. The extents of nick ligation by D540A (open circles), D540S (open squares), D540R (open triangles), and D540K (open diamonds) were plotted as a function of time. (d) Nick-joining activities of Asp540 mutants at each temperature for 120 min. Error bars represent the standard deviation (*n* = 3) [103].

As mentioned in the previous section, thermostable DNA ligases are utilized in LCR and LDR, which require a heat denaturation step. However, thermostable DNA ligases possess weaker ligation activity at lower temperatures (20–40°C), resulting in the decreased efficiency of the LCR and LDR procedures using short probes with low-melting temperatures. Here we present a structure-guided mutational analysis of the hyperthermostable DNA ligase from* P. furiosus*, in order to improve the ligation efficiency with the thermostability [103]. In Section 4.4, we showed that the C-terminal helix in the closed structure observed in PfuLig connects the AdD and the OBD via five polar or ionic interactions (Figure 13(b)). In contrast, the crystal structures of hLigI and SsoLig revealed that the relative arrangements of the OBD and the AdD are quite different from that of PfuLig (Figure 9(a)). Taken together, we hypothesized that the binding efficiency of the DNA ligase to the DNA substrate would increase by reducing the ionic interactions between the AdD and the OBD, resulting in improved ligation efficiency. The ionic residues involved in the interactions between the AdD and the OBD were selected and mutated to create a series of mutants in which certain ionic residues are replaced or deleted. First, the series of the alanine mutants (1ala, 2ala, and 3ala), in which the ionic residues at the C-terminal helix are replaced with alanine residues, were prepared, and the series of deletion mutants (d4, d8, and d15) were also created. Then the initial reaction rates of these mutants were measured at the maximum temperature (60°C). The initial reaction rates were increased according to the number of replaced or deleted ionic residues (Figure 14(a)) [104].

Next, we found that the initial reaction rate was largely improved when the fourth ionic residue (Asp540) from the C-terminus was mutated, in addition to the 3ala mutations (D540A/3ala). Therefore the single alanine mutant of Asp540 (D540A) was created and assessed the effect of the mutation on the ligation efficiency. The reaction rate of D540A was almost the same as that of D540A/3ala [103], suggesting that the effect of the mutation of Asp540 dominates the improvement of the alanine mutations of the ionic residues on the C-terminal helix. Asp540 is located at the AdD-interacting surface of the OBD and forms an ionic pair with Arg414, in the vicinity of the AMP binding site in the AdD (Figure 12).

A series of Asp540 mutants, in which Asp540 was replaced with serine (D540S), lysine (D540K), and arginine (D540R), were also constructed, and their ligation efficiencies were evaluated. As a result, the initial reaction rates of D540K and D540R were similarly the fastest among all of the mutants, followed by D540S, then D540A, and finally the wild type (Figure 14(b)). The evaluation of the ligation efficiencies of these Asp540 mutants over the broad temperature range from 20 to 80°C revealed that D540K and D540R were also the most productive. Thus, we successfully generated the PfuLig mutant with highly improved ligation efficiency over a broad temperature range, while maintaining its useful thermostability, by replacing Asp540 with a basic residue such as lysine or arginine (Figures 14(c) and 14(d)) [103].

Further investigation into the effects of the replacement of Asp540 with a basic residue on the ligation process revealed that the replacement contributed to both the adenylylation and DNA binding steps. These results suggested that the increase in the number of basic residues in the proximity of the active site was advantageous for the adenylylation step, in which the negatively charged pyrophosphate is pulled away from the ATP molecule in the active site pocket and that the alteration to the basic residue was also efficient for the AdD/OBD domain opening, caused by the repulsion between either Arg540 or Lys 540 and Arg414, which formed the ion pair with Asp540 (Figure 12) [103].

## 6. Conclusions

Archaea live in extreme conditions, even in the temperature range of 80°C to 100°C, and have yielded many useful enzymes for gene technology, such as hyperthermostable DNA polymerases and DNA ligases. In this review, we have described the utilization of thermostable DNA ligases in common molecular biology protocols and the structural mechanism of DNA ligase and shown some examples of protein-engineered DNA ligases. Improvements of these enzymes are potentially effective for advancing the existing methods mentioned above and beneficial for constructing novel biotechnological methods. Several protein engineering strategies, such as fusion with other proteins or site-direct mutagenesis based on structural information, may facilitate the construction of application-specific DNA ligases in the future. The enzymes can be improved artificially, based on their three-dimensional structures, even if they have naturally evolved for a long period of time.

As many Archaea thrive in extreme environments, it will be very interesting to learn how the fidelity mechanisms of these extremophilic organisms have adapted to overcome these harsh conditions. Therefore, archaeal enzymes will continue to play an important role in future research and will be employed in numerous aspects of gene technology.
